# Jaw Hyperostosis in Infants: Keep in Mind Caffey’s Disease

**DOI:** 10.5334/jbr-btr.956

**Published:** 2016-01-29

**Authors:** Maria Magalhães, Maria José Noruegas, Conceição Sanches, Manuel Salgado

**Affiliations:** 1 Instituto Português de Oncologia Francisco Gentil Porto, PT; 2 Hospital Pediátrico, Centro Hospitalar e Universitário de Coimbra, PT

**Keywords:** Jaw, Hyperostosis, Caffey disease

A previously healthy 3 month-old female child was referred to the Oncology Department of Hospital Pediátrico de Coimbra with a four day history of right facial swelling. Pain triggered by palpation was the only complaint. There was no effacement of the temporomandibular angle or palpable cervical lymphadenopathy. Laboratory workup revealed anemia (hemoglobin: 9,5 g/dL, N: 11, 1-14, 1), thrombocytosis (617 × 10^3^/uL, N: 200–550), leukocytosis (16.23 × 10^9^/L, N: 4, 0-11, 0), hyperphosphatemia (2,36 mmol/L, N: 1, 25-2.10) and increased erythrocyte sedimentation rate (49 mm/h, N < 13). Alkaline phosphatase levels were normal (275UI/L, N: 145–320).

Facial soft tissue sonography demonstrated a hypoechoic area (arrow) adjacent to the right jaw (dashed arrow), involving the muscular planes; bone surface was regular (Figure 1). Parotid and submandibular glands echogenicity was preserved. Magnetic resonance imaging (Figure 2) A- T1-weighted (W), B- T2-W, C- T2-W with fat suppression, D- contrast-enhanced) showed increased volume of the right half of the mandible (dashed arrow), ipsilateral medial pterygoid and masseter muscles (solid arrow), whose signal on T2-weighted images was increased, indicating edema. Finally, skull radiograph (Figure 3) revealed right jaw hyperostosis (arrow) with adjacent soft tissue swelling (dashed arrow). Soft tissue biopsy showed chronic inflammation, without evidence of neoplastic process; in the bone sample, there was increased trabecular bone, with prominent fibroblastic reaction.

**Figure 1:**
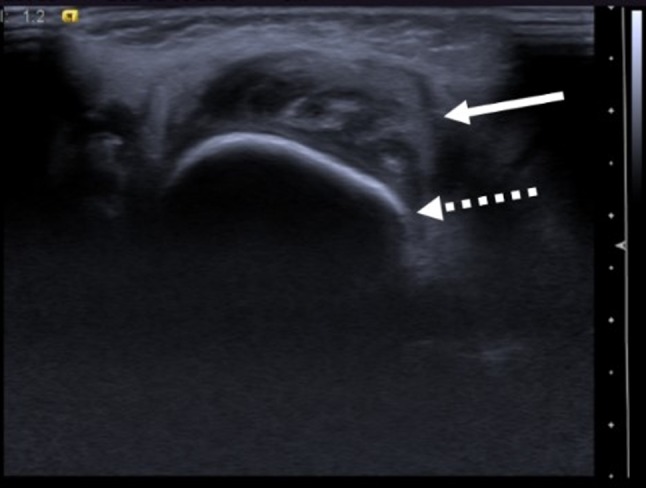


**Figure 2:**
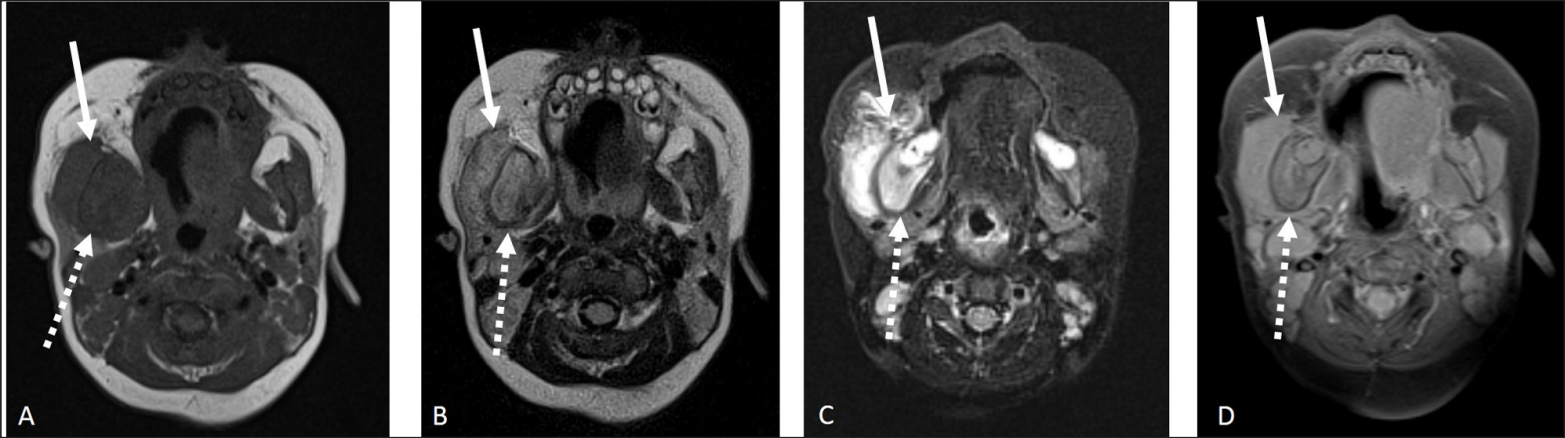


**Figure 3:**
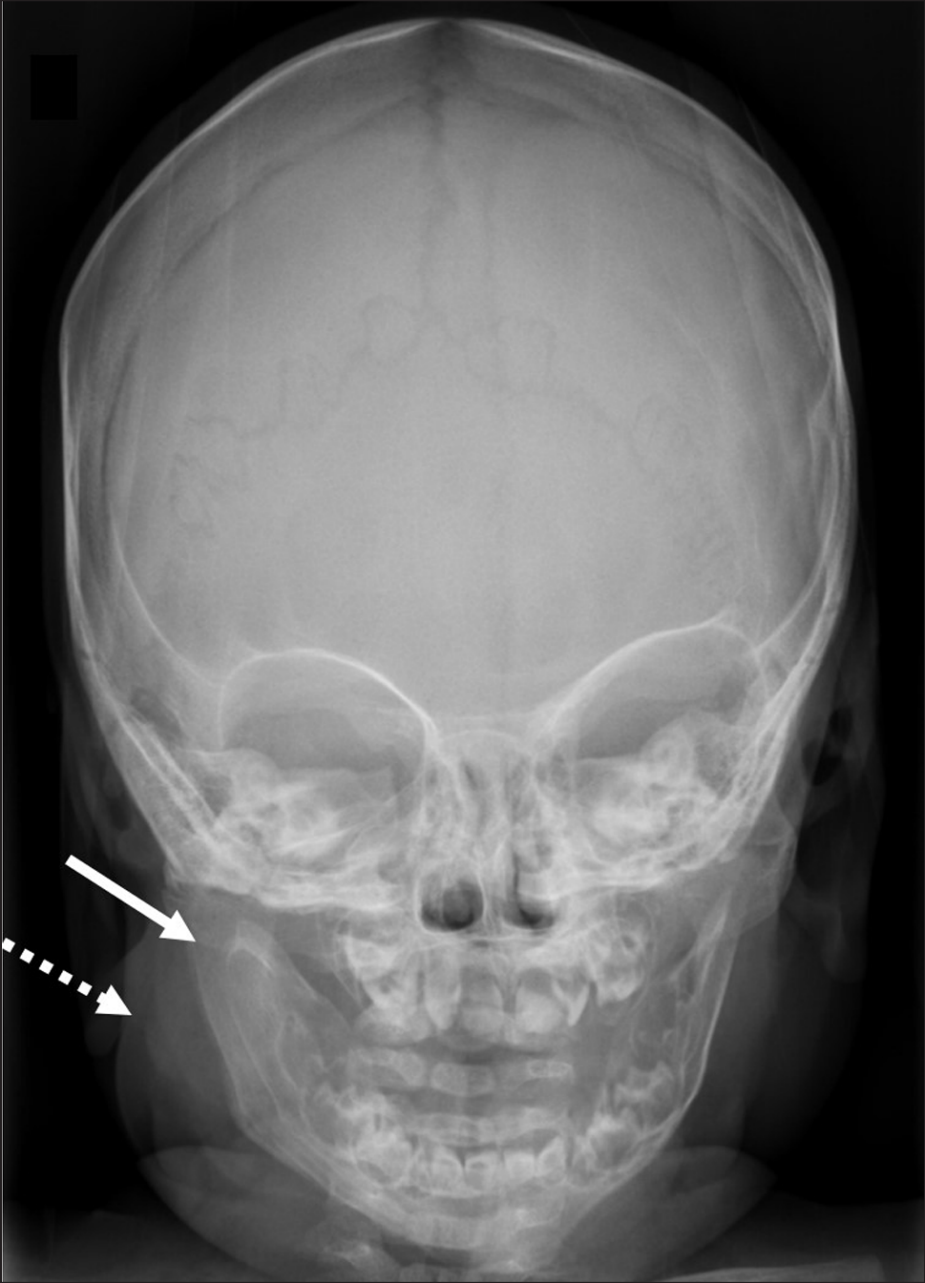


Diagnosis of Caffey’s disease was assumed and the child was treated with ibuprofen (10 mg/kg/3id) during two months. Six months later she had no swelling or other complaints. Molecular genetic testing for *COL1A1* mutation 3040C > T was negative.

## Comment

Caffey’s disease, a self limiting condition affecting young infants, is characterized by acute inflammation of the periosteum and the overlying soft tissue, accompanied by systemic changes (irritability and fever). Although the etiology is not completely understood, familial and sporadic forms appear to exist. A particular mutation in the *COL1A1* gene (3040C > T), the gene encoding the α1 chain of type I collagen, is seen in the autosomal dominant form.

The infant responds as if an infection were present with leukocytosis, limited motion of the involved bone(s) and hyperostosis. Indeed, periosteum is a multifunctional tissue that allows bone to adapt throughout life to mechanical, hormonal and pathological changes. When injured, periosteal new bone is deposited. Most often, this occurs when intramedullary inflammatory, tumoral or blood cells extend through the cortex and irritate the periosteum, but this may happen whenever an adjacent inflammatory process occurs.

In infants there are different causes that can damage periosteum leading to hyperostosis, including bone tumors, osteomyelitis, infection (for instance syphilis), hypervitaminosis A, scurvy, trauma and child abuse. Since malign bone disease is not unusual in children, exclusion of bone tumor should be done.

At radiograph, aggressive periosteal reaction can be seen in bone tumors, but also in some benign conditions such as osteomyelitis. These processes usually present other associated findings which can aid in differential diagnosis, such as regional osteopenia and loss of bony trabecular architecture in the case of osteomyelitis. Clinical and laboratory data are of utmost importance to differentiate the remaining entities and conventional radiography should be in the initial imaging workup, as highlighted in this case. Moreover, initial workup with other imaging studies such as MR, can act as a confounding factor.

Although rare, Caffey’s disease should not be overlooked and should be in the differential diagnosis of childhood bone hyperostosis.

## Competing Interests

The authors declare that they have no competing interests.
